# Chronological Rehabilitation Treatment Varying by Stage for Constructive Disability

**DOI:** 10.3390/clinpract13040084

**Published:** 2023-08-08

**Authors:** Takana Okamoto, Tomoo Mano

**Affiliations:** 1 Division of Central Clinical Laboratory, Nara Medical University Hospital, Kashihara 634-8521, Japan; 2 Department of Rehabilitation Medicine, Nara Medical University, Kashihara 634-8521, Japan; 3 Department of Rehabilitation Medicine, Nara Prefecture General Medical Center, Shichijo-nishimachi, Nara 630-8581, Japan

**Keywords:** constructive disability, rehabilitation, instrumental activities of daily living

## Abstract

Constructive disability poses significant challenges. However, such manifestations are often overlooked. To address these disabilities, customized rehabilitation is necessary across disease stages. This case report demonstrates how customized, stage-based occupational therapy interventions can effectively rehabilitate patients with progressive constructive disability. Herein, a 33-year-old female patient with progressive constructive disability underwent direct training and compensatory therapy in early stages and progressed to instrumental activities of daily living training in later stages. This case demonstrates how such customized, progressive occupational therapy can achieve substantial functional improvements even for patients with advancing cognitive and physical impairments from constructive disability.

## 1. Introduction

“Constructive apraxia” is a condition in which the spatial form of a constructed object is impaired. The term ”constructive disability” is used because constructive apraxia is commonly complicated by cognitive impairment [1,2,3]. It is often overlooked by patients or medical staff [4]. Constructive disability occurs with damage to either the left or right hemisphere; right hemisphere lesions are often accompanied by visuospatial processing deficits [5,6]. There are many cases in which rehabilitation treatment shows limited improvement due to associated deficits in spatial information processing and procedural action processes [7]. Rehabilitation therapy is considered to be effective for movement training that is closer to daily life. Evaluation tasks of constructive disability, such as drawing pictures and arranging building blocks, may not accurately reflect the difficulties experienced in daily activities [1]; thus, evaluation and training that align with the instrumental activities of daily living (IADL) of each individual are critical to ensure appropriate assessment and treatment.

## 2. Case Presentation

The patient was a 33-year-old female with no significant medical history. She lived with her husband and daughter. She previously worked as an aesthetician and was currently on maternity leave. The activities of daily living training (ADL) and IADL were independent, and she was right-hand dominant. She initially presented with generalized seizures, was diagnosed with a right parietal lobe lesion, and had visited our hospital for surgery.

**Figure 1:**
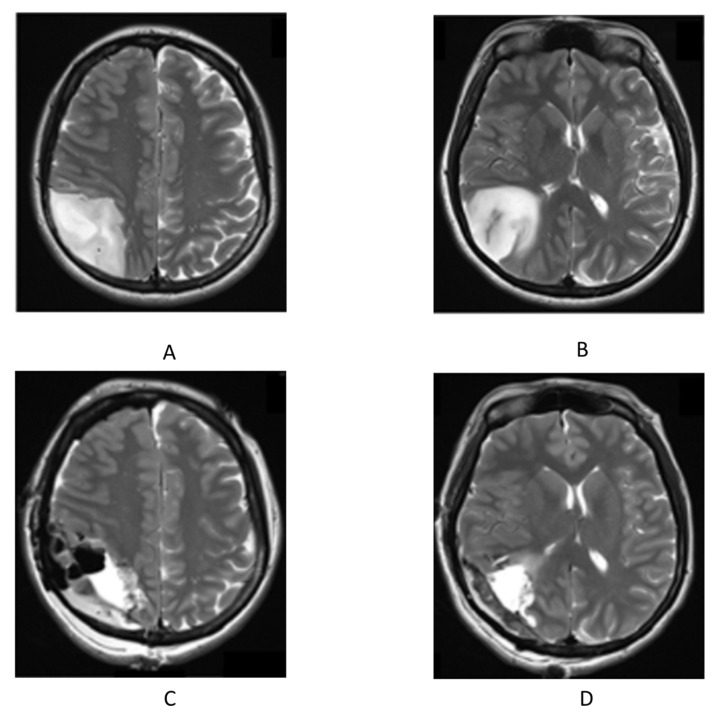
T2-weighted image of head magnetic resonance imaging. (**A**,**B**) before surgery; (**C**,**D**) after surgery.

T2-weighted magnetic resonance imaging (MRI) of the head (Figure 1) revealed a space-occupying lesion with a mass measuring 5.7 × 4.6 × 5.6 cm in her right parietal lobe. T2-weighted image acquisition is used in radio imaging diagnosis [8]. The patient underwent awake lumpectomy under local anesthesia and was pathologically diagnosed with glioblastoma. Postoperatively, oral treatment with temozolomide was initiated in combination with radiotherapy.

### 2.1. Initial Evaluation

**Figure 2:**
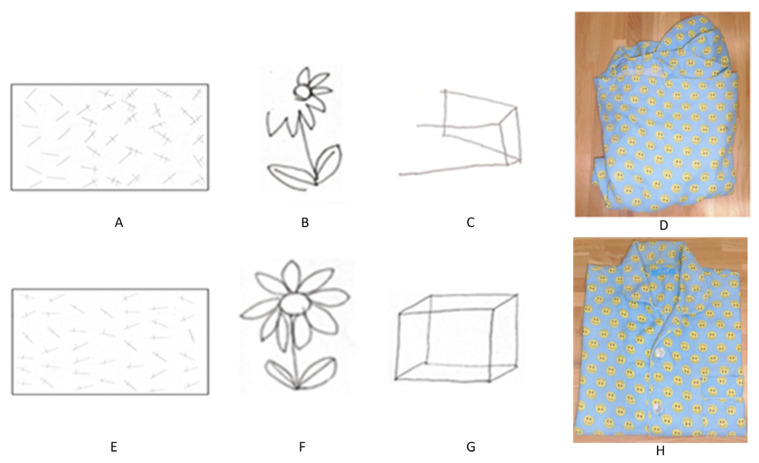
Transition of clinical evaluation. Upper row: initial evaluation (**A**–**D**), lower row: final evaluation (**E**–**H**). (**A**,**E**) Line segment erasure test. (**B**,**F**) Flower copying test. (**C**,**G**) Cube copying test. (**D**,**H**) Completed form of folded clothes.

**Figure 3:**
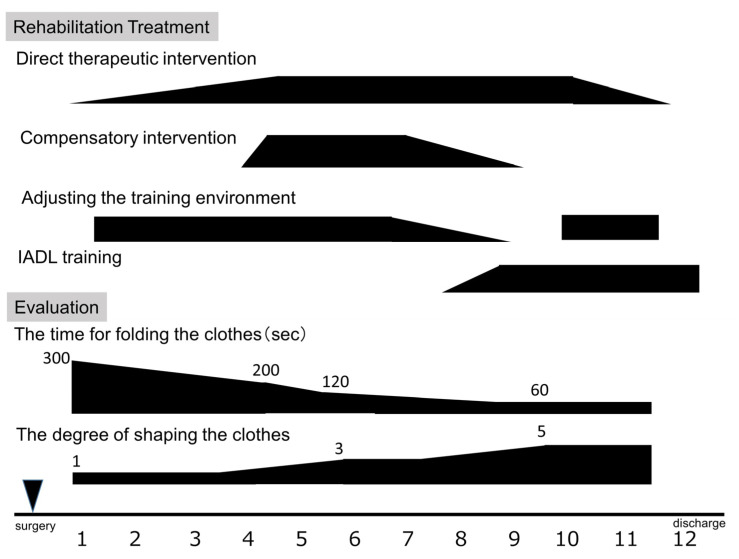
Clinical course of rehabilitation treatment and evaluation. sec, seconds.

The cranial nervous system revealed visual field defects in the lower left quadrant. Her kinesthetic and balance functions were normal. During left gaze, there was a delay in eye movement onset. In reaching movements of the upper extremities, the left upper extremity failed to reach the target in a single movement, with a delay of approximately 2 s compared to the right extremity; a similar result was observed when looking at the left side. The line bisection test was normal, but a trisection of straight lines and circles was unsuccessful. The line-crossing test yielded a score of 23/40, and the flower reproduction test score was 0/1. The course cube test score was 0/131 [8] (Figure 2). The Mini-Mental State Examination (MMSE) score was 29/30 [9], and although there was a reduction in figure copying, there was no obvious decrease in delayed recall. Although the basic movements in the hospital ward were generally independent, the patient often encountered obstacles, including people, without noticing them on her left side while walking. The ADL showed that she had leftover food because of poor recognition of the left side when eating. When changing her jacket, she sometimes did not notice that the buttons were incorrectly fastened or that the collar was folded in. The Functional Independence Measure score was 97, and it consisted of 62 motor and 35 cognitive items. In terms of time recognition, there was a difference between actual time perception and self-recognition. The number of seconds for folding the clothes was measured, and the degree of shaping was defined on a five-point scale (5 out of 5 indicates correctly folded clothing; 1 point was deducted for the following items: wrapping is different, the sleeves are not folded, the hem is not folded, or the shape is distorted). The action of folding clothes took more than 5 min, and the finished form was in a state where the left sleeve and hem were not properly folded and could not be shaped. In particular, errors increased when attention was focused on patterns and pockets, and many procedural errors could not be corrected by verbal guidance alone (Figure 3). In summary, the patient had visuospatial cognitive impairment (construction disorder, visual attention disorder, visual ataxia, sensory hemispatial neglect, etc.) and left visual field impairment [9].

### 2.2. Intervention

Occupational therapy was performed five times a week for 40 min a day (Figure 3). With reference to a previous report [7], cognitive rehabilitation was performed while being conscious of the difficulty level so as not to cause an erroneous reaction. The intervention occurred as follows: (1) direct therapeutic intervention was repeated while confirming procedures and precautions based on memory, and providing feedback; (2) compensatory therapeutic intervention using tactile and kinesthetic senses as visual compensation was performed; and (3) the intervention was conducted in a step-by-step manner. Therefore, specific IADL training was incorporated and the degree of difficulty was adjusted.

Direct therapeutic intervention is a treatment that stimulates and activates impaired function by repetitive practice of the impaired function or ability, and it has been reported that the functional improvement effect of repetitive motion is related to the regeneration and sprouting of neuronal axons. Direct therapeutic intervention is one of the most common types of rehabilitation for constructive impairment.

Compensatory interventions also utilize other functions or factors to achieve the impaired function or ability. Compensatory interventions are reported to reorganize the internal structure of nerve tissue by substituting other remaining healthy functions and combining impaired function with residual function. In addition, compensatory treatments are used to compensate for impaired functions and abilities using external assistance. Furthermore, we aim to promote spatial analysis and integration with various sensory inputs [7].

IADL training consists of setting-based therapeutic interventions. IADL training is aimed at enhancing adaptive behavior in daily life and preventing and reducing problem behaviors. Previous reports have demonstrated that improvement can be observed through a systematic step-by-step approach based on the patient’s specific functionality and abilities [4].

### 2.3. Occupational Therapy Course

(1) Phase 0

Occupational therapy was started after the operation, and bradykinesia, reaching movement, and oculomotor disorder showed immediate improvement. However, there was a decline in time recognition, such as being unable to prepare for the scheduled start time, even if the start time and examination time were verbally communicated on the same morning. In addition, many errors were observed regarding the folding of clothes. A follow-up MRI confirmed a marked improvement in postoperative edema, but no improvement was observed when the clothes were folded.

(2) Phase 1

To address the challenges encountered during daily activities, we began with the patient sitting on their knees and attempting to put on patterned shirts and long-sleeved front-opening shirts with pockets, which is a frequent activity in daily life. However, the presence of visual distractions in the training room hindered her ability to continue working effectively. Considering the effects of visual attention impairment, we adjusted the environment by minimizing visual stimuli on the walls and organizing working material on desks. The patient preferred patterned shirts but changed to a solid color shirt because she thought that the pattern may have distracted her. Due to their difficulty, the patient began occupational therapy with short-sleeved shirts instead of long sleeves. Owing to the difficulty in focusing on her lower left hand, the treatment was performed in a standing position to increase her field of vision while grasping the entire garment. Instructions for the procedures were given orally to her as her auditory comprehension and memory were preserved. She was instructed to manually perform a series of actions, such as checking the front and back, checking the order from the right side when aligning the front body to the center, and checking that the shirt was in the correct position. Regarding awareness of time, the patient was instructed to keep note of the clock, along with staff and family. We provided an opportunity to check the elapsed and remaining time during rehabilitation.

(3) Phase 2

Five weeks after the operation, she increased the speed with which she folded her clothes and was able to complete the task in about 3 min. However, there were many operational errors such as the left half of the clothes being twisted, the sleeves sticking out, and the shoulder width being different between the left and right sides. We questioned the lack of visual insight for predicting the finished form and thought it would be difficult to make judgments based solely on sight. Therefore, we introduced a compensatory intervention that recognizes the discrepancy between tactile and visual sensations to call attention to the correction area. By repeating this training, the patient experienced a reduction in attentional distractions and an improvement in attention distribution. Eight weeks after surgery, she exhibited a noticeable improvement in her ability to independently identify and rectify twisted or protruding sleeves in her clothing. In addition, the patient became aware of the corrections that were required on her left side without prompting and was able to put on long-sleeved and buttoned shirts. Twelve weeks after surgery, her lower left cognitive deficit was correctable with verbal commands.

Time awareness plays a crucial role in enhancing our ability to be punctual and prepared. We emphasized the importance of allocating specific time for rehabilitation treatment and practiced task-switching techniques. Additionally, due to difficulties in perceiving the left side as a whole, the patient was unable to cut food into equal sizes during cooking.

To address this, in the equipartition task, we introduced a compensatory therapeutic intervention in which tactile input was performed in the same manner as the action of folding clothes.

(4) Phase 3

Rehabilitation training was performed while the patient sat on a sofa to create an environment similar to that at home. We aimed to improve the patient’s ability to handle multiple tasks simultaneously by simulating real-life scenarios and implementing them using dialogue. As a result, there was an improvement in the recognition and distribution of attention toward clothing as a whole.

At 10 weeks postoperatively, the patient demonstrated independent time management skills, with a decrease in the frequency at which she checked her watch. Moreover, errors in everyday life decreased, and left-sided cognitive deficits were only observed in certain situations [10].

The patient was discharged after ensuring her competence in daily activities and providing her with appropriate guidance.

### 2.4. Final Evaluation

The scores were 40/40 for the line crossing test, 1/1 for the flower copying test, 1/1 for the cube copying test (Figure 2), and 49/131 for the course cube test. The trisecting-line injury remained mild. Visual field defects remained unchanged.

The MMSE score was 30/30, ADL was independent, and the left side was overlooked. The action of folding clothes could be performed without error within 1 min (Figure 3). The patient was able to independently perform tasks including time management, laundry, shopping, and simple cooking.

## 3. Discussion

In therapeutic intervention, methods of adding verbalization and clues to construction tasks have been reported, but none have been definitively established, and treatment methods differ depending on the technician in charge. In addition, there are many types of training, such as drawing pictures and arranging building blocks, and IADL training is rare.

We report a case in which a customized stage-based occupational therapy intervention was effective for a patient with progressive constructive disability. The patient received direct training and replacement therapy in the early stages and transitioned to instrumental activities of daily living training in the later stages. Such customized progressive occupational therapy was able to achieve substantial functional improvement even in patients with progressive cognitive and physical impairments due to structural impairment.

### 3.1. Impact of Configuration Disorders on Daily Life

Constructive disability refers to a condition that impairs the ability to perceive the spatial relationship of objects and manipulate physical space, significantly impacting daily life activities [1]. Specific ADLs impacted by constructive disability include the inability to fold clothes and bedding properly, complete daily routines in a timely manner, write on one side, and walk straight. In the play scene, even if the class of cards and sequence of numbers are understood, challenges arise in comprehending the sequence progression, combining sequences, and identifying effective strategies.

### 3.2. Evaluation of Constructive Disability

The evaluation of constructive disability requires a comprehensive analysis of the disability elements of both constructive and intellectual disability and their relationship with each other. As the structural impairments may vary between left and right cortical lesions, it is necessary to predict the extent of the impairment based on prior patient information and imaging tests, which is followed by the selection of appropriate evaluation methods. In right cerebral hemisphere disorders such as this case, it is necessary to elucidate the difficulty of visual perceptual and visuospatial analysis of the composition object. Constructive disorders are often evaluated using drawing tasks, two-dimensional puzzles, building block pattern-building tasks, three-dimensional building block-building tasks, and body position-building tasks targeting a specific part of the body. In addition, we evaluated the performance of drawing and specific composition tasks. Characteristic errors observed due to construction tasks include (1) fragmentation and omission, (2) persistence, (3) rotation, (4) replacement and addition, and (5) difficulty in integration and placement. In addition to various characteristic errors that appear in figures and pictures drawn by patients, combined patterns, and three-dimensional objects created, it is also important to observe the patients’ response to the process of solving the task.

### 3.3. Rehabilitation Approaches and Techniques

Herein, IADL training was conducted by adjusting the degree of difficulty, and training efficiency was improved. To reduce incorrect reactions, we established a focused training environment from the start of the intervention. We initiated training with simple clothing items and encouraged awareness of perceiving clothing through visual cues and the challenges encountered during movement. At the beginning of the intervention, poor visual recognition was observed in the structural aspect; however, improvement was observed through repeated practice. In addition, by using tactile and kinesthetic senses, spatial analysis and integration were facilitated by confirming the positional relationship of each component through both visual and tactile kinesthetic senses. In this case, the disability persisted even after the general improvement period of postoperative cerebral edema [11], and IADL training may have played a role in this improvement [12,13]. Direct therapeutic intervention is a bottom–up approach. A bottom–up approach focuses on identifying the underlying causes of configuration disorders through individualized analysis and delivering targeted interventions. This approach is often chosen when intellectual capacity is declining. In this case, it was introduced at an early stage and had the effect of improving the speed of folding clothes. Conversely, compensatory intervention is a top–down approach, requires a broader perspective and insight, and relies on intact intellectual capacity to solve compositional problems, leading to improved folding accuracy. This approach is selected based on the individual’s specific needs and abilities [14].

In this case, the introduction of compensatory training accelerated the improvement, but environmental adjustment from the beginning seems to have been a major factor. To ensure the execution of the configuration task, several methods can be employed to guide patients to a solution, such as by attaching numbers to each component or writing down part of the design or pattern in advance. Another method alters the behavior of a person and follows a temporal execution procedure. Since the state and changes in the environment affect the work and results, environmental adjustment during training may enhance the benefits of rehabilitation treatment [15,16].

On the other hand, rehabilitation programs have limitations, and some patients who do not require our supervision or who have memory impairment do not experience improvement. It is necessary to plan a program for each patient and to assess and review it each time. There are few reports on rehabilitation treatment for structural disorders, so it is necessary to evaluate as many similar cases as possible and discuss what kind of rehabilitation treatment is optimal.

Other methods reported to promote learning include the cue increment method (a method of gradually decreasing the number of cues required to accomplish a task from the maximum, and adjusting the amount of cue decrease appropriately according to the progress of the task) and reverse chaining (usually contrary to the execution procedure of, practice from one step before the final stage and completing the task in the reverse order until it is completed). It is important to select an appropriate method for efficient learning [9].

## 4. Conclusions

Once destroyed, nerve tissue does not regenerate, but training has been reported to reorganize nerve tissue while changing its internal structure. Constructive disorder is a complex higher brain dysfunction; however, it may be improved by performing appropriate rehabilitation at each stage.

## Data Availability

The data that support the findings of this study are available from the corresponding author upon reasonable request.
